# Does the Location of Fat Accumulation Affect the Degree of Aortic and Renal Arterial Calcification?

**DOI:** 10.3390/biomedicines12040860

**Published:** 2024-04-12

**Authors:** Ivan Ordulj, Mirko Tandara, Kristian Jerković, Frano Šarić, Miodrag Beneš, Sanja Lovrić Kojundžić, Maja Marinović Guić, Danijela Budimir Mršić

**Affiliations:** 1 Clinical Department of Diagnostic and Interventional Radiology, University Hospital of Split, Spinčićeva 1, 21000 Split, Croatia; iordulj@gmail.com (I.O.); mirko.tandara@gmail.com (M.T.); jerkovickristian@gmail.com (K.J.); fsaric33@gmail.com (F.Š.); lovric.sanja@gmail.com (S.L.K.); maja.marinovic.guic@gmail.com (M.M.G.); 2 Institute of Public Health Sveti Rok Virovitica, Podravina County, 33000 Virovitica, Croatia; ben7es@gmail.com; 3 School of Medicine, University of Split, Šoltanska 2, 21000 Split, Croatia; 4 University Department of Health Studies, University of Split, Ruđera Boškovića 35, 21000 Split, Croatia

**Keywords:** aorta, renal artery, visceral fat, aortic calcification, atherosclerosis, MSCT

## Abstract

The vascular risk associated with obesity is particularly associated with visceral adiposity, but recent studies suggest that ectopic fat might contribute to the increased risk of atherosclerotic cardiovascular disease. Our study aimed to explore the connection between arterial calcification of the aorta and renal arteries with visceral and ectopic fat deposits, including liver, pancreatic, and renal sinus fat. Retrospective analysis of thoracoabdominal multi-slice computed tomography (MSCT) scans of 302 patients included measurements of calcification volumes of thoracic and abdominal aorta, and of both renal arteries. On the same scans, the visceral fat volume, liver-to-spleen ratio, pancreatic-to-spleen ratio, and both renal sinus fat areas were retrieved. Logistic regression showed the left kidney sinus fat area to be the most strongly associated with calcifications in the aorta and both renal arteries (coef. from 0.578 to 0.913, *p* < 0.05). The visceral fat positively predicted aortic calcification (coef. = 0.462, *p* = 0.008), and on the contrary, the pancreatic fat accumulation even showed protective effects on thoracic and abdominal aorta calcification (coef. = −0.611 and −0.761, *p* < 0.001, respectively). The results suggest that ectopic fat locations differently impact the calcification of arteries, which should be further explored.

## 1. Introduction

Atherosclerosis is a chronic inflammatory disease that is characterized by the formation of plaques within arterial walls. It is initiated by intimal lesions due to lipid retention and deposition accompanied by smooth muscle cell and fibrous matrix proliferation, which gradually develop into the formation of a plaque that can become calcified [1,2]. Atherosclerotic cardiovascular disease and its complications are a common cause of death, especially in the Western countries [1]. The most commonly affected arteries are coronary, carotid, and peripheral arteries, resulting in myocardial infarction, acute ischemic stroke, and peripheral arterial disease, respectively [3,4,5].

Calcification of the arterial wall has been shown to predict increased cardiovascular risk, independent of the classic cardiovascular risk factors [6,7,8,9]. Arterial calcification consists of calcium salt precipitates, mostly in apatite form, similar to the hydroxyapatite found in bone. In the past, vascular calcification was seen as an inert endpoint of atherosclerosis. However, recently it has become clear that it is an actively regulated process already occurring in the early stages of atherosclerotic lesions [10,11,12]. Arterial calcification is seen in studies using various imaging techniques [13]. However, they are best visualized with computed tomography (CT). Due to signal voids on magnetic resonance imaging (MRI), calcification is difficult to analyze on MRI scans. Using CT, calcium within plaque is usually suspected when a pixel exceeds 130 Hounsfield units (HU), and patchy arterial wall calcification is typical sign of atherosclerosis. In the coronary arteries, this technique is currently the only validated technique for the quantification of vascular calcification [14]. Other arterial calcifications are encountered incidentally as a part of chest and abdominal CT studies, or as a part of dedicated scans, such as renal angiography, evaluation of patients with an embolic event, or preprocedural assessments. So far, the amount, extent, and interrelation of aortic and renal arteries’ calcifications have not commonly been investigated.

Several risk factors associated with the presence or progression of vascular calcification have been identified [6,7,8,9,10,15], among which age, hypertension, and obesity play important roles. Obesity-related health risks are usually associated with the accumulation of visceral abdominal fat in contrast to subcutaneous deposits of fat. However, the development of obesity also leads to ectopic adipose tissue storage [16], such as in the liver, pancreas, perivascular tissue, renal sinus, skeletal muscles, and in other locations. Ectopic fat accumulation can increase the risk of cardiovascular disease and lead to dysfunction of organs in which it accumulates [17]. The normal renal sinus contains a small amount of adipose tissue that lines other structures within it. Through excess deposition of adipose tissue in the renal sinus, compression of various renal structures may occur, especially the inner medulla, which, unlike the entire kidney, is not protected by the fibrous capsule [18]. It has been previously shown that renal sinus adipose tissue may have an independent association with renal functions [19,20]. However, renal sinus adipose tissue research is limited to only a couple of recently published studies. Foster et al. [20] showed that renal sinus adipose tissue is associated with an increased risk of hypertension and chronic kidney disease (CKD). Apart from arterial atherosclerotic calcification accumulation in the intimal layer, CKD is also related to medial calcification, independent of hyperlipidemia and other classical atherosclerotic risk factors [21].

The main goal of our study was to explore the possible relation of visceral and various ectopic fat accumulations, including renal sinus, liver, and pancreatic fat deposits, on the calcification of aorta and renal arteries using thoracoabdominal CT scans. Furthermore, the extent of thoracic and abdominal aortic calcification, and calcification of renal arteries, was investigated and compared.

## 2. Materials and Methods

### 2.1. Study Design and Participants

The study is retrospective and cross-sectional. It was conducted on randomly selected adult patients who underwent both urgent thoracic and abdominopelvic CT scans requested from the Surgical Emergency Department between April 2020 and June 2023. Inclusion criteria: patients older than 18 years at the time of the scanning, patients with detailed medical history and complete imaging data. Exclusion criteria: incomplete medical history; inadequate imaging documentation, such as only thoracoabdominal scanning without pelvic scans, or lack of pre-contrast multi-slice computed tomography (MSCT) scans of any region; splenectomy or serious parenchymal organ injury. The age and sex of the patients were retrieved from the Hospital Information System (HIS). Imaging data were retrospectively extracted from Picture Archiving and Communication System (PACS). The CT scanning was performed using a 128-slice CT, Somatom, Siemens, Munich, Germany.

### 2.2. Vascular Calcification Measurement

**Figure 1:**
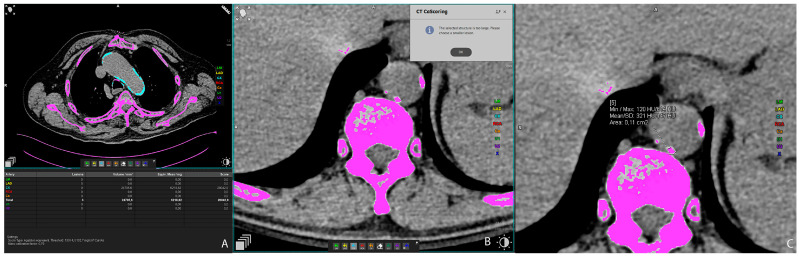
Representative axial CT images showing arterial calcification measurement using syngo.via postprocessing software VB60A_HF08 (**A**), and freehand ROI measurement of calcification volume (**B**,**C**).

Vascular calcifications were measured by trained radiologists using the Siemens (Munich, Germany) Syngovia post-processing tool “Ca Scoring”, which estimates calcifications on native CT scans. The tool is routinely used for coronary artery calcifications with Agatston score calculation. Each researcher passed training prior to the measurements. The procedure was as follows: on the native/pre-contrast scans, each calcification of the thoracic and abdominal aorta and both renal arteries was manually labeled (Figure 1A) and added up to the total volume of calcification of each artery. If the vascular calcifications were too close to the bone structures (spine) or were extending into an adjacent artery (for example thoracic into abdominal aorta), the freehand regions of interest (ROIs) on all scans where the lesion extended were used to delineate calcifications of interest and calculate calcification volume (Figure 1B,C). Finally, the volume of calcification within each artery was expressed in mm^3^ [22].

### 2.3. Visceral Fat Volume Measurement

**Figure 2:**
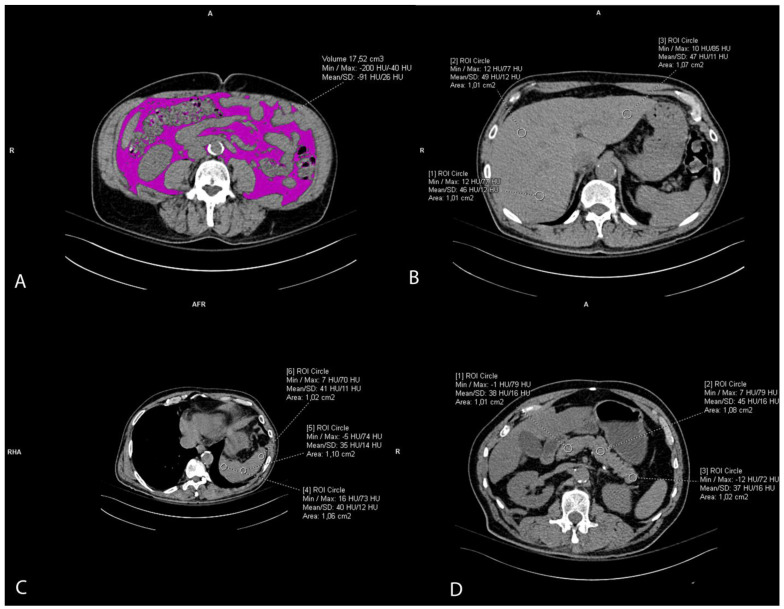
Representative axial CT images showing visceral fat volume estimation (**A**), liver attenuation measurement (**B**), spleen attenuation measurement (**C**), and pancreas attenuation measurement (**D**).

The visceral fat volume, expressed in cm^3^, was measured on the native/pre-contrast CT scans of the abdomen and pelvis semiautomatically. First, at the umbilical level, a line at the inner contour of the abdominal muscles was carefully manually drawn. The Hounsfield unit of fat tissue attenuation from −200 and −40 HU was automatically segmented within this area surrounded by the manually drawn line. Finally, the visceral fat volume was automatically calculated by the Siemens syngo.via VB60A_HF08 software (Figure 2A). The intraclass correlation coefficient (ICC) was 0.982 (95% CI 0.96–0.99).

### 2.4. Ectopic Fat Accumulation Measurements

Liver attenuation was measured by drawing three round regions of interest (ROI) of the approximately same size, 1 cm^2^, at the portal vein level representing HU. The ROI was placed within the left lobe, right anterior lobe, and right posterior lobe of the liver. All ROIs were carefully distributed in the liver parenchyma, excluding the biliary, vascular, and other structures. (Figure 2B). The used liver attenuation was an average of the three HU at the mentioned measurement sites. Spleen attenuation was very similarly measured by averaging three manually placed round ROIs (approximately 1 cm^2^), representing HU, at three different areas of spleen on the same slice (Figure 2C). After the liver and spleen attenuation measurements, the liver-to-spleen attenuation ratio was calculated by dividing the average liver with average spleen attenuation. The index was used as a measure of ectopic fat accumulation in the liver parenchyma (liver steatosis). Pancreas attenuation measurement was similarly performed using ROI at the pancreatic parenchyma at the uncinate process, and the head, neck, body, and tail of the pancreas (Figure 2D). An average of the HU in the pancreas was calculated, and then the pancreas-to-spleen attenuation ratio was calculated in the same manner, by dividing the mean attenuation values of the pancreas and spleen. The pancreas-to-spleen attenuation was used as a measure of ectopic pancreatic fat accumulation (pancreatic steatosis). The ICC for the liver-to-spleen ratio was 0.939 (95% CI 0.80–0.97), and for the pancreas-to-spleen ratio it was 0.852 (95% CI 0.71–0.93).

### 2.5. Renal Sinus Fat Accumulation and Renal Imaging Parameters

**Figure 3:**
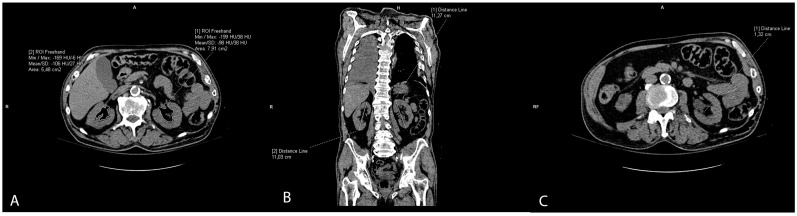
Representative CT scans showing renal parameter volume measurements: renal sinus fat area (**A**), length of the kidney (**B**), and renal parenchymal thickness (**C**).

The slices of the renal hilus of each kidney containing the largest amount of sinus fat were used to measure a renal sinus fat area in cm^2^, representing ectopic renal sinus fat accumulation. The sinus fat surface area was traced manually and identified by its low-density fatty content with a negative HU (Figure 3A). The length of the kidney was measured on the coronal reconstructions as the longest craniocaudal diameter of each kidney (Figure 3B). Renal parenchymal thickness was measured at the hilar level on axial scans (Figure 3C).

### 2.6. Laboratory Parameters

In terms of laboratory parameters, the following kidney function markers were collected from HIS: creatinine and estimated glomerular filtration rate. These parameters were obtained on the same day as the MSCT scanning.

### 2.7. Statistical Analysis

Statistical analysis was performed using the programming language Python ver. 3.9, and the Pingouin ver. 0.5.3, SciPy ver. 1.11.3, Scikit—learn ver. 1.0.1 [23], and Statsmodels ver. 0.13.1 [24] statistical libraries were also used. For visualization, we used Seaborn ver. 0.12.2 [25]. Correlations included different fat measurements and arterial calcification volumes of the aorta and renal arteries, and these were calculated using Spearman’s correlation. Due to a substantial number of zero values in the dependent variable for the abdominal, thoracic, and renal arteries, logistic regression was employed. Robust linear regression was utilized for the variables “Right sinus kidney fat area” and “Left sinus kidney fat area”, as they contained numerical values without many zeros. Alpha (α) for all regressions was 0.05. To standardize the data and to ensure that the regression coefficients were on a comparable scale, StandardScaler was employed. By standardizing the data using StandardScaler, each feature was transformed to have a mean of 0 and a standard deviation of 1. Consequently, the regression coefficients represent standardized regression coefficients. The initial predictors in all regressions were as follows: age, sex, smoking, hypertension, diabetes, cholesterol, eGFR, visceral fat, L/S, P/S, right kidney sinus fat area, left kidney sinus fat area, creatinine. Subsequently, the variance inflation factor (VIF) was computed for each predictor to mitigate multicollinearity. Predictors were chosen such that no VIF exceeded 5. Predictors with *p* values of statistical significance were retained, while those far from the alpha value were removed. The final set of predictors comprises those that are statistically significant with low VIF values (VIF < 5).

## 3. Results

**Figure 4:**
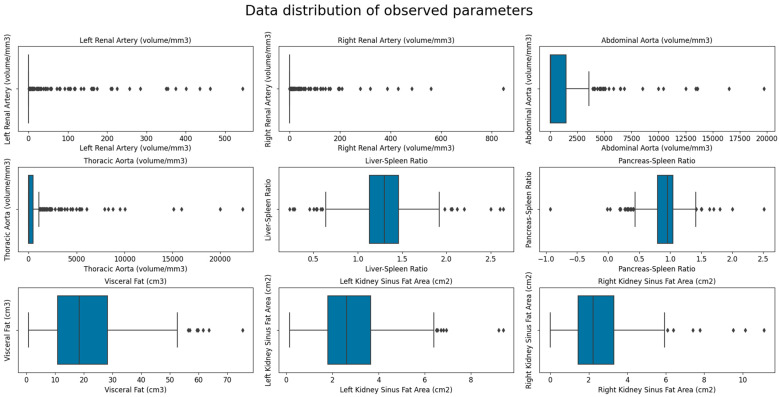
Box plot showing the data distribution of the observed parameters—measures of visceral and ectopic (L/S, P/S and RSFA) fat accumulation and calcification volumes of the thoracic aorta, abdominal aorta, and both renal arteries.

Baseline patients’ data showed that the mean age of the 302 participants included was 49.47 ± 17.76 years, and 28.15% of patients were women. Mean liver, spleen, and pancreas attenuations were within the normal ranges (52.18, 40.99, and 37.46 HU, respectively). The mean volume of visceral fat was 20.37 cm^3^, the mean L/S was 1.29, and the P/S was 0.92. Both kidneys were of normal size and parenchymal thickness (Table 1). The mean creatinine serum level was 86.29 μmol/L, and the estimated glomerular filtration rate (eGFR) was 87.84 ± 22.78, which indicates stage 2 chronic kidney disease. The most heavily calcified vessel was the abdominal aorta (1198.32 mm^3^) followed by the thoracic aorta (867.81 mm^3^) and renal arteries (left 22.60 mm^3^ and right 21.71 mm^3^). Figure 4 shows the data distribution of the observed parameters, measures of visceral and ectopic fat accumulation (liver-to-spleen ratio, pancreas-to-spleen ratio, and renal sinus fat) and the calcification volumes of the thoracic aorta, abdominal aorta, and both renal arteries.

**Figure 5:**
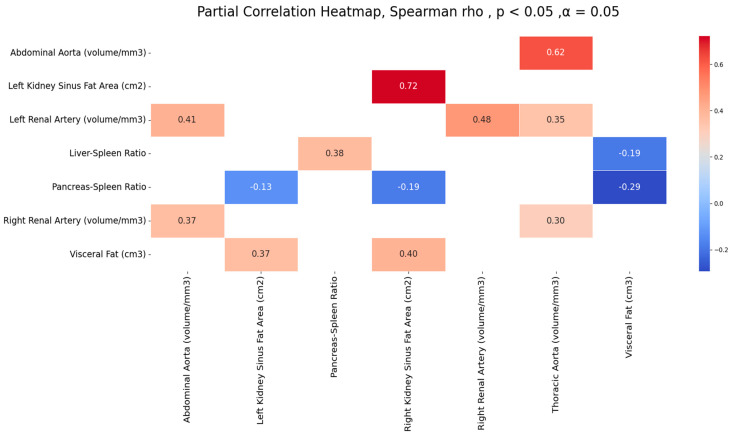
Significant correlations among observed arteries’ calcification volumes and measures of fat accumulation.

Table 2 and Figure 5 present significant correlations among measures of abdominal fat accumulation (visceral fat, liver-to-spleen ratio, pancreas-to-spleen ratio, renal sinus fat) and the calcification of the aorta and renal arteries. Positive strong to moderate correlations were observed for the calcification of arteries among themselves; the highest correlation was of the thoracic and abdominal aortic calcification volumes (r = 0.62) and the calcification of the left and right renal arteries (r = 0.47). Similarly, positive moderate correlations were observed between the visceral fat and renal sinus fat area (r = 0.40 and 0.37), and among two ectopic fats, L/S, and P/S (r = 0.37). A negative correlation was observed between visceral fat and liver-to-spleen ratio and pancreas-to-spleen ratio, and renal sinus fat and pancreas-to-spleen ratio. However, no significant correlation was observed between any one measure of ectopic fat and the calcification of observed arteries.

The logistic regression (Regression I, Table 3) covered all observed parameters, including demographic, laboratory, and MSCT parameters in relation to aortic calcification. Independent predictors of thoracic aortic calcification were age (coef. = 2.951), non-smoker status (coef. = −0.593), and visceral fat (coef. = 0.542), together explaining 56.4% of thoracic calcification. Independent predictors of abdominal aortic calcification were age (coef. = 3.687) and liver-to-spleen ratio (coef. = −0.472), together explaining 64.4% of abdominal calcification.

The second regression analysis (Regression II, Table 3) included only abdominal fat parameters in the regression model to explore their relation to aortic calcification, which altogether explained 17.5% and 20.2% variance of the thoracic and abdominal artery calcification volumes, respectively. In general, the strongest independent predictors of aortic calcification were pancreas-to-spleen ratio (coef. = −0.611 for thoracic and −0.761 for abdominal aorta) and fat area of the left sinus kidney (coef.= 0.913 for thoracic and 0.857 for abdominal aorta), while visceral fat showed a somewhat lower prediction (coef. = 0.462) for the thoracic aorta, as in Table 3.

Similarly, another logistic regression was performed which aimed to explore the prediction of observed parameters, i.e., demographic, laboratory, and MSCT parameters, on the calcification of left and right renal arteries (Regression I, Table 4). The regression showed that age was an independent positive predictor for both renal arteries (coef.= 2.170 for the right and coef. = 1.976 for the left renal artery). In the case of the right renal artery, sex was an independent, but weaker, predictor (coef. = 0.594), and for the left renal artery it was visceral fat (coef. = 0.511), as in Table 4, Regression I. Other parameters did not show significant independent prediction.

The second logistic regression (Regression II, Table 4) included only abdominal fat parameters in a regression model to explore their relation to calcification of both renal arteries. The results showed that ectopic and visceral fat deposits explained 10.3% of the right renal artery and 13.6% of the left renal artery’s calcification variance. The highest prediction for both renal arteries among the fat tissue variables showed a left renal sinus fat area (coef. = 0.695 and 0.578 for right and left renal artery), followed by the visceral fat (coef. = 0.492 for left renal artery), as in Table 4, Regression II.

## 4. Discussion

Our study showed no significant correlation between either measure of ectopic fat or calcification of observed arteries; however, logistic regression revealed the significant positive association of left renal sinus fat with the calcification of all four arterial locations, in comparison to right renal sinus fat. Visceral fat, liver-to-spleen ratio, and pancreas-to-spleen ratio were also independent predictors for some arteries, and pancreas-to-spleen ratio showed a small but protective effect on the aorta. Furthermore, strong correlations between thoracic and abdominal aortic calcifications, as well as between left and right renal artery calcification, were observed. The most heavily calcified vessel was the abdominal aorta, followed by the thoracic aorta and renal arteries, with a negligible difference between the left and right renal arteries. Finally, it is important to notice that our CT measurements showed high ICC, >0.9, since CT measurements theoretically might be affected by an operator-associated bias. According to previous research, arterial calcification usually begins at the abdominal aorta and spreads cranially to the thoracic aorta and caudally to iliac vessels. The process can also involve aortic branches, such as renal vessels or anterior visceral branches, although this is less well known. Therefore, we analyzed correlations among calcifications of renal arteries and thoracic and abdominal aorta to reveal if their involvement differs and to what extent. The strong correlation between the calcification volume in the thoracic and abdominal aorta is in accordance with a study by Maruyama et al. [26], who found a strong correlation between the calcification scores of the aortic arch and the abdominal aorta. We found several papers concerning the CT quantification of renal artery calcifications [27,28,29,30,31]. As in previous studies [27,28,29,32], renal artery calcifications were significantly associated with older age. There was no statistical difference between calcifications in the left and right renal artery, which is in accordance with Yang H et al. [32], who found no statistical difference in the incidence of renal artery plaque between the left renal artery and right renal artery.

The most intriguing finding of our study is a significant positive association of left renal sinus fat with calcifications of all four arteries, in comparison to right sinus fat, which was not associated with either artery. Our study showed that the renal sinus fat volume of the left kidney was somewhat larger than that of the right kidney (2.83 cm^2^ vs. 2.56 cm^2^), and this result was consistent with previous studies [33,34,35]. Previous studies have shown that renal fat sinus deposition was related to other deleterious fat depots, such as liver steatosis and visceral fat [33,36,37,38,39], similar to our results. Zhang et al. [33] found that there were correlations between the renal sinus fat in both kidneys with visceral adipose fat, hepatic fat fraction, and pancreatic fat fraction. Adipose tissue located in different body compartments may play distinct roles and contribute to comorbidities through unique pathophysiological mechanisms [40]. The renal calyces, renal pelvis, and blood vessels are surrounded by varied amounts of adipose tissue in the renal sinus. In the metabolically benign condition, renal sinus fat was beneficial to glomerular cells, and it may promote regeneration and anti-fibrosis effects, and reduce the release of pro-inflammatory factors by endothelial cells and podocytes. However, in a metabolically malignant condition, excessive fat accumulation in the renal sinus would result in increased intraabdominal pressure and compression of structures within the renal sinus, which increases renal hydrostatic pressure and activates the renin–angiotensin–aldosterone system (RAAS). Activation of the RAAS promotes hypertension, insulin resistance, atherosclerosis, and other adverse physiological effects related to obesity [41,42]. In addition, as an ectopic perivascular fat, renal sinus fat has the paracrine effect of secreting inflammatory cytokines and vasoconstrictive factors, which could lead to local inflammation, oxidative stress, lipotoxicity and fibrosis [20]. Findings from the Framingham Heart Study have revealed that renal sinus fat was related to multiple cardiometabolic risk factors [38]. It was found that renal sinus fat, as a perivascular fat depot in close contact with the adventitia of large, medium, and small arteries, has unique features that differ from other fat depots, and undergoes changes early in the development of metabolic diseases [37]. Krievina et al. [34] found that the renal sinus fat was directly related to the early renal injury markers Skim-1 and FGF-21. Lee et al. [43] revealed that both metabolic syndrome and obesity were associated with lower renal sinus fat attenuation index. A randomized controlled trial demonstrated that increased renal sinus fat was directly associated with hypertension [44]. Recently, it was also found that the accumulation of renal sinus fat seems to be involved in the pathogenesis of hypertension in obesity, and following bariatric surgery, loss of renal sinus fat was associated with remission from hypertension [45]. The reason may be that the asymmetric accumulation of RSF could be result of anatomical differences between the left and right renal veins [34]. The right renal vein receives blood from the right kidney only, but the left renal vein receives blood from the left renal vein as well as the left gonadal and adrenal veins [46]. The average left renal blood flow demonstrated a significant reduction relative to the average right renal blood flow. Additionally, there existed a substantial variation in the orientation of the calyces, given that the angles of the calyces in the right and left renal organs exhibit notable dissimilarities [47]. A potential hypothesis for the notable increase in renal sinus fat accumulation in the left kidney is the presence of an innate structural phenomenon that may result in asymmetrical blood flow to the renal organs [34].

Another finding was a negative correlation of pancreatic steatosis with other fat deposits, and its negative association with calcification of the abdominal and thoracic aorta. While pancreas volume decreases with age, pancreatic adipose content tends to increase [48,49]. Indeed, pancreatic steatosis is mainly due to pancreatic fatty infiltration rather than intracellular accumulation [50]. Nevertheless, events leading from triglyceride accumulation to lipid peroxidation are similar, so that the term nonalcoholic steatopancreatitis (NASP) has been coined [51]. Due to its inflammatory asset, NASP is likely to have crucial consequences. Pancreatic steatosis has been suggested as a predisposing factor for atherosclerosis, as it correlates with the carotid–femoral pulse wave velocity [52]; however, we suggest it might even have a minor “protective” effect on the vascular calcifications, maybe through disturbing normal insulin metabolism and its association with insulin resistance.

In conclusion, the study shows that age’s predominant effect on the extent of arterial calcification is much stronger than the cumulative effect of several abdominal fat deposits. Generally, a single fat deposit is relatively weakly related to arterial calcification compared to age. Also, readers must be aware that there are no direct and immediate clinical implications of these results. However, MSCT is an excellent method for accurately quantifying the calcification of each artery, and for separating and measuring every fat deposit in the body. We believe the study opens new insights into the pathophysiology of arterial calcification, which is important for improving knowledge and understanding of the atherosclerosis process. Finally, the study has some limitations. The study’s retrospective and cross-sectional design prevented us from more deeply understanding the relationships. Second, not all ectopic deposits of fat were taken into account due to a technical inability to estimate them on CT scans and current postprocessing software. The MSCT scans of polytrauma patients were chosen in the study, since it is only in this population that native/pre-contrast multi-slice CT scans (thorax, abdomen, pelvis) are routinely performed, which allowed the whole aorta to be scanned and analyzed. However, in this particular setting, the laboratory risk factors for atherosclerosis, such as LDL-cholesterol levels, were not routinely collected as they are not relevant for trauma management. Similarly, in the injured patient, the stress response can provoke acute hyperglycemia, and, therefore, the values of blood glucose levels were not taken into account in the regression analyzes. The MSCT cannot differentiate the calcification location (intimal vs. medial), so the study could not precisely differentiate if the calcifications that were found were atherosclerotic or CKD-related.

Apart from the mentioned limitations, the study has several strengths. It included a relatively high number of patients with no significant chronic comorbidities since they had CT scanning due to an urgent surgical indication, such as trauma. The independent effects of four ectopic intrabdominal fat deposits were analyzed. Also, the calcification volumes in the whole aorta were measured and compared to the calcifications of both renal arteries, which is not often investigated, and might help to better understand the extent and pattern of calcifications in different vessels.

## 5. Conclusions

To conclude, the calcification of the thoracic and abdominal aorta and both renal arteries show a moderate to strong correlation. Some ectopic and visceral fat measurements are associated with arterial calcifications, especially left renal sinus fat, which should be investigated further.

## Figures and Tables

**Table 1:** Baseline, clinical, and observed parameters in the study population sample.

Parameters	Mean ± SD
No. of participants (n)	302 (100%)
Age (years)	49.47 ± 17.76
Sex (n, %)	women 85 (28.15%)
	men 217 (71.85%)
Smoking	
Yes	61 (20.19%)
No	105 (34.76%)
Ex	26 (8.60%)
Not avalable	110 (36.42%)
Arterial hypertension, yes (n, %)	58 (19.20%)
Diabetes, yes (n, %)	25 (8.27%)
Cholesterol, yes (n, %)	38 (12.58%)
Visceral fat (cm^3^)	20.37 ± 12.60
Liver attenuation (HU)	52.18 ± 10.71
Spleen attenuation (HU)	40.99 ± 5.95
Liver-to-spleen ratio	1.29 ± 0.32
Pancreatic attenuation (HU)	37.46 ± 11.37
Pancreatic-to-spleen ratio	0.92 ± 0.30
Left kidney length (cm)	11.17 ± 1.24
Right kidney length (cm)	10.92 ± 1.30
Left kidney parenchymal thickness (cm)	1.73 ± 0.29
Right kidney parenchymal thickness (cm)	1.72 ± 0.59
Left kidney sinus fat area (cm^2^)	2.83 ± 1.53
Right kidney sinus fat area (cm^2^)	2.56 ± 1.60
Creatinine (μmol/L)	86.29 ± 29.49
eGFR (mL/min/1.73m^2^)	87.84 ± 22.78
Thoracic aorta calcification volume (mm^3^)	867.81 ± 2566.46
Abdominal aorta calcification volume (mm^3^)	1198.32 ± 2642.19
Left renal artery calcification volume (mm^3^)	22.60 ± 74.46
Right renal artery calcification volume (mm^3^)	21.71 ± 82.13

Abbreviations: HU—Hounsfield Units, eGFR—estimated glomerular filtration rate.

**Table 2:** Significant correlations among observed variables of fat tissue accumulation and the aorta and renal arteries.

Variable 1	Variable 2	r	CI 95%	*p*
AA	TA	0.62	[0.54 0.69]	<0.0001
LRA	RRA	0.47	[0.38 0.56]	<0.0001
LRA	AA	0.40	[0.3 0.5]	<0.0001
RRA	AA	0.37	[0.26 0.47]	<0.0001
LRA	TA	0.34	[0.24 0.45]	<0.0001
RRA	TA	0.30	[0.19 0.41]	<0.0001
LKSFA	RKSFA	0.72	[0.66 0.77]	<0.0001
VF	RKSFA	0.40	[0.3 0.5]	<0.0001
VF	LKSFA	0.37	[0.26 0.47]	<0.0001
L/S	P/S	0.37	[0.27 0.47]	<0.0001
P/S	VF	−0.29	[−0.4 −0.18]	<0.0001
L/S	VF	−0.18	[−0.3 −0.07]	<0.0001
P/S	RKSFA	−0.18	[−0.3 −0.07]	<0.0001
P/S	LKSFA	−0.13	[−0.25 −0.01]	<0.0001

Abbreviations: TA—thoracic aortic calcification volume, AA—abdominal aortic calcification volume, RRA—right renal artery calcification volume, LRA—left renal calcification volume, P/S—pancreas-to-spleen ratio, L/S—liver-to-spleen ratio, VF—visceral fat, RKSFA—right kidney sinus fat area, LKSFA—left kidney sinus fat area.

**Table 3:** Logistic regression showing significant predictions of thoracic and abdominal aortic calcification volumes with all other variables (Regression I) and with fat tissue measurements (Regression II).

	Thoracic Aorta Calcification Volume (mm^3^)			Abdominal Aorta Calcification Volume (mm^3^)		
I. Regression	Coefficient	95% CI	*p* Value	Coefficient	95% CI	*p* Value
Age	2.951	2.282; 3.621	<0.05	3.687;	2.854; 4.530	<0.05
Visceral fat	0.542	0.074; 1.010	<0.05	/	/	/
Non-smoker status	−0.593	−0.994; −0.194	<0.05	/	/	/
L/S	/	/	/	0.472	0.921; −0.023	<0.05
	R^2^ = 0.564			R^2^ = 0.644		
II. Regression	Coefficient	95% CI	*p* Value	Coefficient	95% CI	*p* Value
Visceral fat	0.462	0.122; 0.803	0.008	0.317	−0.025; 0.659	0.069
L/S	0.279	−0.030; 0.589	0.077	0.099	−0.212; 0.411	0.532
P/S	−0.611	−0.986; −0.237	0.001	−0.761	−1.171; 0.352	<0.0001
RKSFA	−0.297	−0.808; 0.212	0.253	−0.011	−0.567; 0.544	0.968
LKSFA	0.913	0.412; 1.415	<0.0001	0.857	0.331; 1.383	0.001
	R^2^ = 0.175			R^2^ = 0.202		

Abbreviations: L/S—liver-to-spleen ratio, P/S—pancreas-to-spleen ratio, RKSFA—right kidney sinus fat area, LKSFA—left kidney sinus fat area.

**Table 4:** Logistic regression showing calcification volumes of both renal arteries in relation to all other parameters (regression I.) and in relation to fat tissue measurements (regression II.).

	Right Renal Artery Calcification Volume (mm^3^)			Left Renal Artery Calcification Volume (mm^3^)		
I. Regression	Coefficient	95% CI	*p* Value	Coefficient	95% CI	*p* Value
Age	2.170	1.572; 2.769	<0.0001	1.976	1.406; 2.548	<0.0001
Sex	0.594	0.235; 0.954	0.001	/	/	/
Visceral fat	/	/	/	0.511	0.180; 0.843	0.002
	R^2^ = 0.324			R^2^ = 0.350		
II. Regression	Coefficient	95% CI	*p* Value	Coefficient	95% CI	*p* Value
Visceral fat	0.315	−0.042; 0.673	0.084	0.492	0.137; 0.848	0.007
L/S	0.199	−0.107; 0.506	0.202	0.122	−0.188; 0.434	0.775
P/S	−0.124	−0.437; 0.189	0.436	−0.283	−0.618; 0.051	0.097
RKSFA	−0.131	−0.606; 0.344	0.589	−0.116	−0.595; 0.361	0.632
LKSFA	0.695	0.209; 1.182	0.005	0.578	0.091; 1.067	0.020
	R^2^ = 0.103			R^2^ = 0.136		

Abbreviations: P/S—pancreas-to-spleen ratio, L/S—liver-to-spleen ratio, RKSFA—right kidney sinus fat area, LKSFA—left kidney sinus fat area.

## Data Availability

Upon reasonable request.

## References

[1] Ross R. (1999). Atherosclerosis—An inflammatory disease. N. Engl. J. Med..

[2] Libby P., Ridker P.M., Hansson G.K. (2011). Progress and challenges in translating the biology of atherosclerosis. Nature.

[3] Naghavi M., Libby P., Falk E., Casscells S.W., Litovsky S., Rumberger J., Badimon J.J., Stefanadis C., Moreno P., Pasterkamp G. (2003). From vulnerable plaque to vulnerable patient: A call for new definitions and risk assessment strategies. Part I. Circulation.

[4] Naghavi M., Libby P., Falk E., Casscells S.W., Litovsky S., Rumberger J., Badimon J.J., Stefanadis C., Moreno P., Pasterkamp G. (2003). From Vulnerable Plaque to Vulnerable Patient: A Call for New Definitions and Risk Assessment Strategies: Part II. Circulation.

[5] Spagnoli L.G., Mauriello A., Sangiorgi G., Fratoni S., Bonanno E., Schwartz R.S., Piepgras D.G., Pistolese R., Ippoliti A., Holmes D.R. (2004). Extracranial thrombotically active carotid plaque as a risk factor for ischemic stroke. JAMA.

[6] Iribarren C., Sidney S., Sternfeld B., Browner W.S. (2000). Calcification of the aortic arch: Risk factors and association with coronary heart disease, stroke, and peripheral vascular disease. JAMA.

[7] Taylor A.J., Bindeman J., Feuerstein I., Cao F., Brazaitis M., O’Malley P.G. (2005). Coronary calcium independently predicts incident premature coronary heart disease over measured cardiovascular risk factors: Mean three-year outcomes in the Prospective Army Coronary Calcium (PACC) project. J. Am. Coll. Cardiol..

[8] Arad Y., Goodman K.J., Roth M., Newstein D., Guerci A.D. (2005). Coronary calcification, coronary disease risk factors, C-reactive protein, and atherosclerotic cardiovascular disease events: The St. Francis Heart Study. J. Am. Coll. Cardiol..

[9] Shaw L.J., Raggi P., Schisterman E., Berman D.S., Callister T.Q. (2003). Prognostic value of cardiac risk factors and coronary artery calcium screening for all-cause mortality. Radiology.

[10] Demer L.L., Tintut Y. (2003). Mineral exploration: Search for the mechanism of vascular calcification and beyond: The 2003 Jeffrey M. Hoeg Award lecture. Arterioscler. Thromb. Vasc. Biol..

[11] Abedin M., Tintut Y., Demer L.L. (2004). Vascular calcification: Mechanisms and clinical ramifications. Arterioscler. Thromb. Vasc. Biol..

[12] Reslerova M., Moe S.M. (2003). Vascular calcification in dialysis patients: Pathogenesis and consequences. Am. J. Kidney Dis..

[13] Rennenberg R.J., Kessels A.G., Schurgers L.J., Van Engelshoven J.M., De Leeuw P.W., Kroon A.A. (2009). Vascular calcifications as a marker of increased cardiovascular risk: A meta-analysis. Vasc. Health Risk Manag..

[14] Kopp A.F., Ohnesorge B., Becker C., Schröder S., Heuschmid M., Küttner A., Kuzo R., Claussen C.D. (2002). Reproducibility and accuracy of coronary calcium measurements with multi-detector row vs. electron-beam CT. Radiology.

[15] Newman A.B., Naydeck B.L., Sutton-Tyrrell K., Feldman A., Edmundowicz D., Kuller L.H. (2001). Coronary artery calcification in older adults to age 99: Prevalence and risk factors. Circulation.

[16] Bjorndal B., Burri L., Staalesen V., Skorve J., Berge R.K. (2011). Different adipose depots: Their role in the development of metabolic syndrome and mitochondrial response to hypolipidemic agents. J. Obes..

[17] Lim S., Meigs J.B. (2014). Links between ectopic fat and vascular disease in humans. Arterioscler. Thromb. Vasc. Biol..

[18] Lamacchia O., Nicastro V., Camarchio D., Valente U., Grisorio R., Gesualdo L., Cignarelli M. (2011). Para- and perirenal fat thickness is an independent predictor of chronic kidney disease, increased renal resistance index and hyperuricaemia in type-2 diabetic patients. Nephrol. Dial. Transplant..

[19] Chughtai H.L., Morgan T.M., Rocco M., Stacey B., Brinkley T.E., Ding J., Nicklas B., Hamilton C., Hundley W.G. (2010). Renal sinus fat and poor blood pressure control inmiddle-aged and elderly individuals at risk for cardiovascular events. Hypertension.

[20] Foster M.C., Hwang S.J., Porter S.A., Massaro J.M., Hoffmann U., Fox C.S. (2011). Fatty kidney, hypertension, and chronic kidney disease: The Framingham heart study. Hypertension.

[21] Rogers M., Goettsch C., Aikawa E. (2013). Medial and intimal calcification in chronic kidney disease: Stressing the contributions. J. Am. Heart Assoc..

[22] Ordulj I., Šarić F., Tandara M., Jerković K., Lovrić Kojundžić S., Marinović Guić M., Beneš M., Budimir Mršić D. (2023). Visceral and Ectopic Abdominal Fat Effect on the Calcification of the Abdominal Aorta and Its Branches-An MSCT Study. Life.

[23] Pedregosa F., Varoquaux G., Gramfort A., Michel V., Thirion B., Grisel O., Blondel M., Prettenhofer P., Weiss R., Dubourg V. (2011). Scikit-learn: Machine learning in Python. J. Mach. Learn. Res..

[24] Seabold S., Perktold J. Statsmodels: Econometric and statistical modeling with python. Proceedings of the 9th Python in Science Conference.

[25] Waskom M.L. (2021). Seaborn: Statistical data visualization. J. Open Source Softw..

[26] Maruyama N., Higuchi T., Ono M., Oguma H., Nakamura Y., Utsunomiya K., Akiya Y., Horikami T., Yamazaki T., Okawa E. (2019). Correlation between Aortic Calcification Score and Biochemical Parameters in Hemodialysis Patients. Contrib. Nephrol..

[27] Tolkin L., Bursztyn M., Ben-Dov I.Z., Simanovsky N., Hiller N. (2009). Incidental renal artery calcifications: A study of 350 consecutive abdominal computed tomography scans. Nephrol. Dial. Transplant..

[28] Siegel C.L., Ellis J.H., Korobkin M., Dunnick N.R. (1994). CT-detected renal arterial calcification: Correlation with renal artery stenosis on angiography. Am. J. Roentgenol..

[29] Allison M.A., Lillie E.O., DiTomasso D., Wright C.M., Criqui M.H. (2007). Renal artery calcium is independently associated with hypertension. J. Am. Coll. Cardiol..

[30] Gayard P., Garcier J.M., Boire J.Y., Ravel A., Perez N., Privat C., Lucien P., Viallet J.F., Boyer L. (2000). Spiral CT quantification of aorto-renal calcification and its use in the detection of atheromatous renal artery stenosis: A study in 42 patients. Cardiovasc. Intervent. Radiol..

[31] Moynahan K., Yoshino M.T. (1993). Aortic and renal atherosclerotic calcifications seen on computed tomography of the spine. A positive predictor of hypertension. Investig. Radiol..

[32] Yang H., Yang R. (2023). The renal artery-aorta angle associated with renal artery plaque: A retrospective analysis based on CT. BMC Med. Imaging.

[33] Zhang Q.H., Chen L.H., An Q., Pi P., Dong Y.F., Zhao Y., Wang N., Fang X., Pu R.W., Song Q.W. (2023). Quantification of the renal sinus fat and exploration of its relationship with ectopic fat deposition in normal subjects using MRI fat fraction mapping. Front. Endocrinol..

[34] Krievina G., Tretjakovs P., Skuja I., Silina V., Keisa L., Krievina D., Bahs G. (2016). Ectopic Adipose Tissue Storage in the Left and the Right Renal Sinus is Asymmetric and Associated with Serum Kidney Injury Molecule-1 and Fibroblast Growth Factor-21 Levels Increase. eBioMedicine.

[35] Lin P., Min Z., Wei G., Lei H., Feifei Z., Yunfei Z. (2020). Volumetric evaluation of renal sinus adipose tissue on computed tomography images in bilateral nephrolithiasis patients. Int. Urol. Nephrol..

[36] Doğan E., Bacaksızlar Sarı F. (2022). Is Fat Deposition of Renal Sinus a Concomitant Finding to Fatty Liver Disease? The First Study Regarding the Relationship Between Kidney and Liver Fat Content with Non-Contrast Computed Tomography. Spartan Med. Res. J..

[37] Notohamiprodjo M., Goepfert M., Will S., Lorbeer R., Schick F., Rathmann W., Martirosian P., Peters A., Müller-Peltzer K., Helck A. (2020). Renal and renal sinus fat volumes as quantified by magnetic resonance imaging in subjects with prediabetes, diabetes, and normal glucose tolerance. PLoS ONE.

[38] Lee J.J., Pedley A., Hoffmann U., Massaro J.M., Levy D., Long M.T. (2018). Visceral and Intrahepatic Fat Are Associated with Cardiometabolic Risk Factors Above Other Ectopic Fat Depots: The Framingham Heart Study. Am. J. Med..

[39] Couch C.A., Fowler L.A., Goss A.M., Gower B.A. (2022). Associations of renal sinus fat with blood pressure and ectopic fat in a diverse cohort of adults. Int. J. Cardiol. Cardiovasc. Risk Prev..

[40] Ferrara D., Montecucco F., Dallegri F., Carbone F. (2019). Impact of different ectopic fat depots on cardiovascular and metabolic diseases. J. Cell Physiol..

[41] Ott C.E., Navar L.G., Guyton A.C. (1971). Pressures in static and dynamic states from capsules implanted in the kidney. Am. J. Physiol..

[42] Dwyer T.M., Mizelle H.L., Cockrell K., Buhner P. (1995). Renal sinus lipomatosis and body composition in hypertensive, obese rabbits. Int. J. Obes. Relat. Metab. Disord..

[43] Lee E.J., Cho N.J., Kim H., Nam B., Jeon J.S., Noh H., Han D.C., Kim S.H., Kwon S.H. (2022). Abdominal periaortic and renal sinus fat attenuation indices measured on computed tomography are associated with metabolic syndrome. Eur. Radiol..

[44] Zelicha H., Schwarzfuchs D., Shelef I., Gepner Y., Tsaban G., Tene L., Yaskolka Meir A., Bilitzky A., Komy O., Cohen N. (2018). Changes of renal sinus fat and renal parenchymal fat during an 18-month randomized weight loss trial. Clin. Nutr..

[45] Moritz E., Dadson P., Saukko E., Honka M.J., Koskensalo K., Seppälä K., Pekkarinen L., Moriconi D., Helmiö M., Salminen P. (2022). Renal sinus fat is expanded in patients with obesity and/or hypertension and reduced by bariatric surgery associated with hypertension remission. Metabolites.

[46] Anjamrooz S.H., Azari H., Abedinzadeh M. (2012). Abnormal patterns of the renal veins. Anat. Cell Biol..

[47] Miller J., Durack J.C., Sorensen M.D., Wang J.H., Stoller M.L. (2013). Renal calyceal anatomy characterization with 3-dimensional in vivo computerized tomography imaging. J. Urol..

[48] Pham Y.H., Bingham B.A., Bell C.S., Greenfield S.A., John S.D., Robinson L.H., Eissa M.A. (2016). Prevalence of pancreatic steatosis at a pediatric tertiary care center. South. Med. J..

[49] Saisho Y., Butler A.E., Meier J.J., Monchamp T., Allen-Auerbach M., Rizza R.A., Butler P.C. (2007). Pancreas volumes in humans from birth to age one hundred taking into account sex, obesity, and presence of type-2 diabetes. Clin. Anat..

[50] Pinnick K.E., Collins S.C., Londos C., Gauguier D., Clark A., Fielding B.A. (2008). Pancreatic ectopic fat is characterized by adipocyte infiltration and altered lipid composition. Obesity.

[51] Mathur A., Marine M., Lu D., Swartz-Basile D.A., Saxena R., Zyromski N.J., Pitt H.A. (2007). Nonalcoholic fatty pancreas disease. HPB.

[52] Ozturk K., Dogan T., Celikkanat S., Ozen A., Demirci H., Kurt O., Turker T., Yilmaz Y., Uygun A. (2018). The association of fatty pancreas with subclinical atherosclerosis in nonalcoholic fatty liver disease. Eur. J. Gastroent. Hepatol..

